# Oculoectodermal syndrome: twentieth described case with new manifestations*

**DOI:** 10.1590/abd1806-4841.20164409

**Published:** 2016

**Authors:** Daniela de Almeida Figueiras, Deborah Maria de Castro Barbosa Leal, Valter Kozmhinsky, Marina Coutinho Domingues Querino, Marina Genesia da Silva Regueira, Maria Gabriela de Morais Studart

**Affiliations:** 1 Instituto de Medicina Integral Professor Fernando Figueira (IMIP) – Recife (PE), Brazil

**Keywords:** Astigmatism, Dermoid Cyst, Ecchymosis, Hemangioma, Hyperpigmentation, Hypopigmentation

## Abstract

Oculoectodermal syndrome is a rare disease characterized by the association of aplasia cutis congenita, epibulbar dermoids, and other abnormalities. This report describes the twentieth case of the disease. We report a 4-year-old female child who presented with the classical features of the syndrome: aplasia cutis congenita and epibulbar dermoids. Our case expands the clinical spectrum of the disease to include: diffuse hyperpigmentation (some following the Blaschko´s lines); hypopigmented skin areas on the trunk; arachnoid cyst on the right fronto-parietal border; rounded left side of the hippocampus; and dermoid cyst underlying the bulb-medullary transition. Our patient also reported infantile hemangioma on the right wrist and verrucous hemangioma on the left leg, the latter not previously described in the literature.

## INTRODUCTION

Oculoectodermal syndrome was first reported in 1993 by Toriello *et al.* Characteristic clinical signs of the syndrome include aplasia cutis congenita and epibulbar dermoids.^1^ Other abnormalities involving the central nervous system, bones, urogenital system and vascular system were also observed. Fifteen cases reviewed by Ardinger *et al.* included giant cell granuloma of the jaw, nonossifying fibroma, cutaneous hyperpigmentation, arachnoid cyst, coarctation of the aorta, and rhabdomyosarcoma.^2^ Studies also report the presence of epidermal nevi following the lines of Blaschko, hypopigmented lesions, and digital anomalies. Oculoectodermal syndrome can be considered a milder variation of encephalocraniocutaneous lipomatosis, differing primarily in the absence of cranial defects. The genetic cause of the disease is unknown and all cases reported were sporadic. Currently, the only detected molecular pathology has been a deletion on Xq12, but its status as a disease-causing aberration remains unclear.^4^ We report a newly diagnosed case with a broad clinical spectrum, with some comorbidities that were described for the first time.

## CASE REPORT

**Figure 1:**
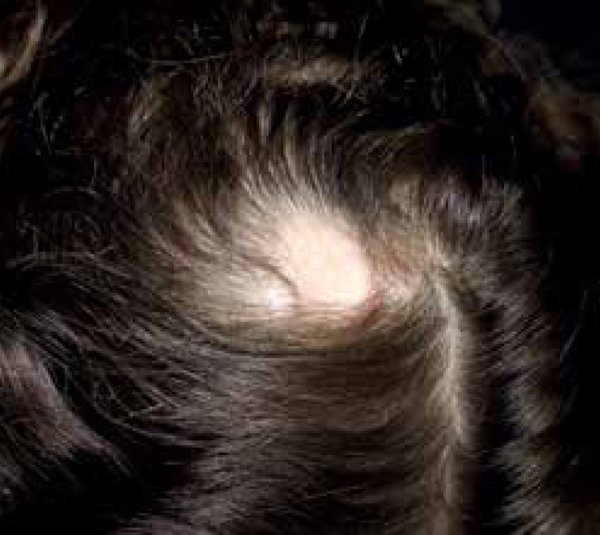
Oval alopecia plaque 3.0x2. 5cm in size on the vertex of the scalp to the right of the midline

**Figure 2:**
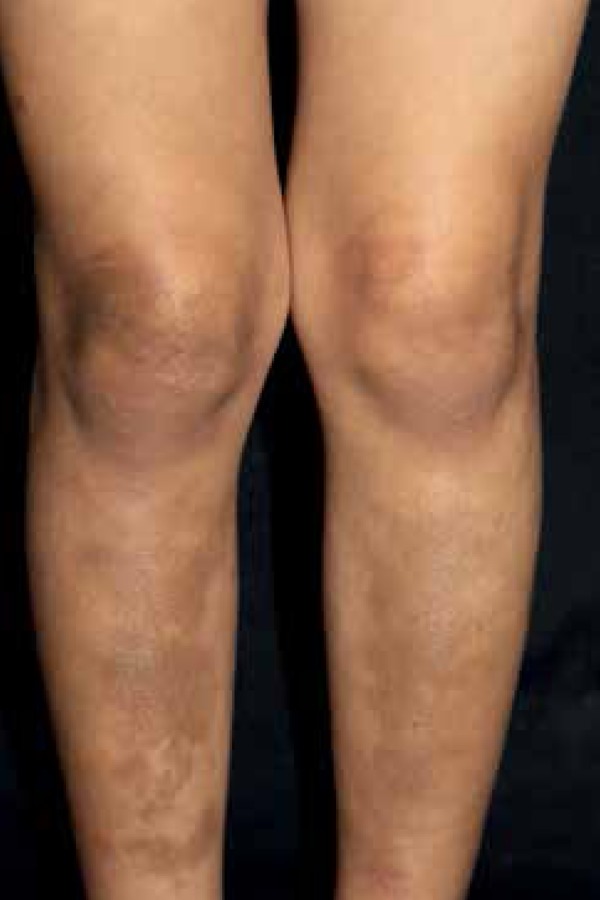
Irregular and a s ymmetr i c residual hyperchromic spots on the lower limbs

**Figure 3:**
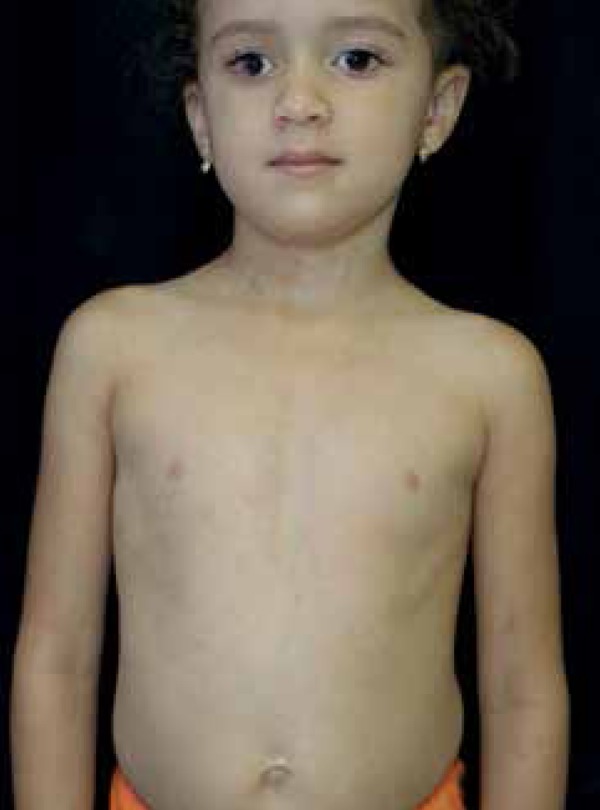
Hyperpigmentation on the neck and trunk following the lines of Blaschko with sharp demarcation at the midline. Epibulbar dermoid on the right eye is also noted, coincident with the side of aplasia cutis.

**Figura 4:**
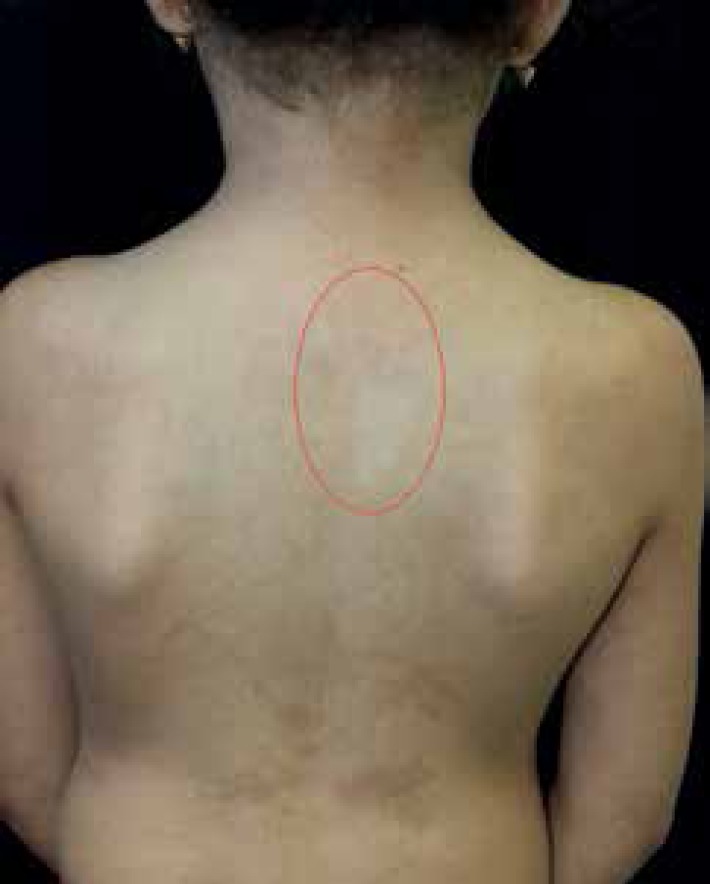
Oval hypopigmentation area highlighted in red, 3.5 x 1 cm in size, jagged edges on the posterior trunk. Hyperpigmentation area following lines of Blaschko on the posterior trunk in the lower back region.

**Figure 5:**
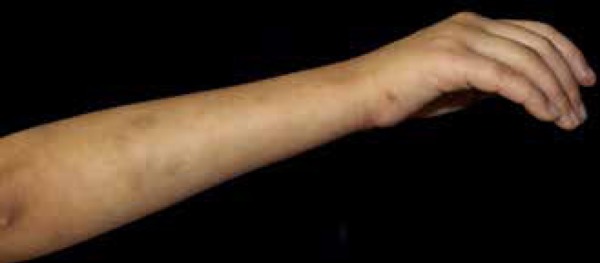
0.5x0.5cm angiomatous lesion on the medial border of the right hand. Residual hyperpigmented patches on the right upper limb.

**Figure 6:**
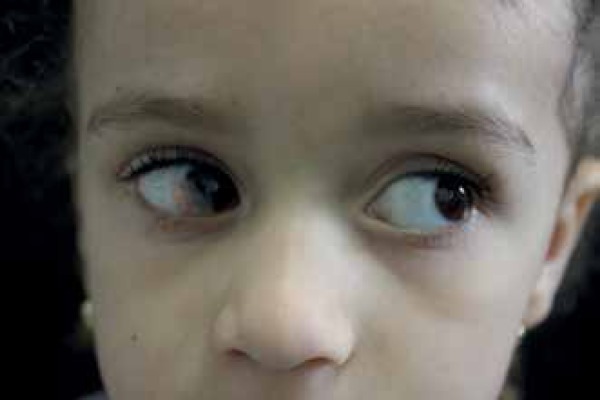
Lesion in the conjunctiva and cornea of the right eye consistent with dermoid cyst

The patient was seen for the first time when she was four. She came from the Recife Eye Institute for dermatologic evaluation of cutaneous hyperpigmentation with history of verrucous hemangioma, alopecia on the scalp, and epibulbar dermoids. The patient is the only child of outbred parents, born by breech delivery with no neonatal or prenatal abnormalities reported. Intellectual ability and postnatal growth were normal. At birth, the baby presented with a “blister” on the right vertex of the scalp, which resolved spontaneously leaving an alopecia plaque 3.0x2.5cm in size, compatible with aplasia cutis diagnosis (Figure 1). Clinical examination revealed residual cutaneous hyperpigmentation on the limbs and flat lesions following path of Blaschko lines on the trunk and neck (Figures 2 and 3). We identified oval-shaped hypopigmentation patches on the posterior trunk, as well as livedo reticularis and tendency to ecchymosis (Figure 4). The patient presented with an angiomatous lesion 0.5x0.5 cm in size on the right wrist and history of verrucous hemangioma on the left lower limb excised at 3 years of age (Figure 5). Ophthalmologic evaluation of the right eye revealed tumor involving the conjunctiva and cornea with significant increase in astigmatism, compatible with dermoid cyst (Figure 6). Magnetic resonance imaging (MRI) of the brain showed lesion compatible with dermoid cyst underlying the bulb-medullary transition, arachnoid cyst on the right fronto-parietal border, rounded left side of the hippocampus and lateral ventricular asymmetry – left ventricle was slightly increased compared to contra-lateral ventricle. Electrocardiogram showed no changes.

## DISCUSSION:

Aplasia cutis congenita (ACC) is a heterogeneous entity that may occur as an isolated defect or as part of a syndrome. The same can be said for epibulbar dermoids. However, the combination of the two abnormalities is rare. In 1993, Toriello *et al.* first described oculoectodermal syndrome (OES). The disease is characterized by ACC, conjunctival dermoid and other anormalidades.^1^ Ardinger *et al.* reported two new patients and reviewed 13 previous cases. They presented evidence that OES is a mild variant of encephalocraniocutaneous lipomatosis (ECCL), differing primarily by lack of intracranial pathology.^2^ Other abnormalities include the central nervous system (arachnoid and dermoid cysts, arteriovenous fistula, hemispheric asymmetries, hypodense periventricular lesions, and ventricular dilation), bones (skull defects, bone cysts), urogenital system (bladder exstrophy and epispadias), and vascular system (aortic coarctation and occlusive syndromes).

Our patient presented had multisystem involvement and other abnormalities never described in OES, such as verrucous hemangioma on the left lower limb, reticular livedo, and a tendency to ecchymosis. Lees M. *et al.* reported a case of OES in a child presented with astigmatism and hemangioma on the right wrist, both present in our report. However, no cases involving verrucous hemangioma were previously reported.^3^

Aslan D. *et al.* described the first case of hypopigmented lesions on the back in unrelated areas of hyperpigmentation. Therefore, our patient confirms that this fact could not be mere coincidence.^4^

Epibulbar dermoid is a choristoma containing a combination of fat, hair follicles and sebaceous glands in the conjunctiva or limbus. OES may manifest itself by unilateral or bilateral epibulbar dermoid. Other signs of the disease include upper eyelid marks, corneal opacity, abnormality in the optic nerve or retina, microphthalmia, and astigmatism. Our patient had a dermoid on the right eye and significant astigmatism.

ACC is defined as a congenital localized absence of skin and is most commonly found on the scalp. OES is characterized by unique or multiple lesions with alopecia and other conditions, such as excess hair growth around aplastic areas, trichorrhexis, and blonde hair coloring. The present case had ACC on the right vertex.

We observed several skin changes: Blaschko lines, hyper-pigmentation, craniofacial lipoma, hyperkeratotic lesions, papules, preauricular pits, epidermal nevus syndrome, linear yellow plaque, and hypopigmentation on the trunk. Our patient displayed hyper-pigmented flat lesions along the lines of Blaschko that occurred during her first year of life, irregular and asymmetrical residual hyperchromia on the limbs, oval hypopigmentation on the back, reticular livedo, and a tendency for ecchymosis.

Despite the changes described in the cranial MRI, our patient had intellectual development consistent with her age, in contrast to some reports of attention deficit disorder, speech disturbances, and developmental disorders described in OES.^5^

Currently, the underlying molecular defect in OES is unknown and all reported cases have been sporadic. Although a new mosaic deletion at Xq12 was recently defined, it is unclear whether this genetic abnormality is pathogenic. Hypotheses suggested for its etiology include autosomal recessive inheritance, tumor suppressor gene mutation, genetic defect in transcription factor that controls eye development, de novo mutation, and mosaicism.^6-9^

Authors suggest that OES is a mild variant of CCLE, a neurocutaneous syndrome that differs primarily by the lack of intracranial lipomas. According to the criteria revised by Moog, our patient seems to have OES, since no intracranial lipoma was observed.^10^

Although skin and eye findings are both relatively innocuous, other systemic diseases – such as seizures, psychomotor retardation, vaso-occlusive crisis, cardiac malformations, and various birth defects – should be considered. We emphasize the importance of seeking a definitive diagnosis when faced with a multisystem disease.
